# Performance investigation of epilepsy detection from noisy EEG signals using base-2-meta stacking classifier

**DOI:** 10.1038/s41598-024-61338-2

**Published:** 2024-05-11

**Authors:** Torikul Islam, Redwanul Islam, Monisha Basak, Amit Dutta Roy, Md. Adil Arman, Samanta Paul, Oleksii Shandra, Sk. Rahat Ali

**Affiliations:** 1 Department of Biomedical Engineering (BME), New Jersey Institute of Technology, Newark, NJ USA; 2 Department of Biomedical Engineering (BME), Khulna University of Engineering & Technology, Khulna, 9230 Bangladesh; 3 Department of Biomedical Engineering (BME), Florida International University, Miami, FL USA; 4 Department of Biomedical Engineering (BME), University of Cincinnati, Cincinnati, OH USA

**Keywords:** EEG, Epilepsy, Base-2-Meta stacking classifier, Machine learning, Feature extraction, Feature ranking, Epilepsy, Biomedical engineering

## Abstract

Epilepsy is a chronic neurological disease, characterized by spontaneous, unprovoked, recurrent seizures that may lead to long-term disability and premature death. Despite significant efforts made to improve epilepsy detection clinically and pre-clinically, the pervasive presence of noise in EEG signals continues to pose substantial challenges to their effective application. In addition, discriminant features for epilepsy detection have not been investigated yet. The objective of this study is to develop a hybrid model for epilepsy detection from noisy and fragmented EEG signals. We hypothesized that a hybrid model could surpass existing single models in epilepsy detection. Our approach involves manual noise rejection and a novel statistical channel selection technique to detect epilepsy even from noisy EEG signals. Our proposed Base-2-Meta stacking classifier achieved notable accuracy (0.98 ± 0.05), precision (0.98 ± 0.07), recall (0.98 ± 0.05), and F1 score (0.98 ± 0.04) even with noisy 5-s segmented EEG signals. Application of our approach to the specific problem like detection of epilepsy from noisy and fragmented EEG data reveals a performance that is not only superior to others, but also is translationally relevant, highlighting its potential application in a clinic setting, where EEG signals are often noisy or scanty. Our proposed metric DF-A (Discriminant feature-accuracy), for the first time, identified the most discriminant feature with models that give A accuracy or above (A = 95 used in this study). This groundbreaking approach allows for detecting discriminant features and can be used as potential electrographic biomarkers in epilepsy detection research. Moreover, our study introduces innovative insights into the understanding of these features, epilepsy detection, and cross-validation, markedly improving epilepsy detection in ways previously unavailable.

## Introduction

Epilepsy is a prominent neurological disease that is denoted by repetitive and unprovoked seizures^1,2^. Seizures result from abnormal sudden electrical discharges in the brain, often typically presenting with loss of consciousness, convulsion, atypical movements, behavioral alterations or emotions^3,4^. Additionally, epilepsy is one of the most common neurological diseases since around 50 million people worldwide have it^5^.

The detection of epilepsy remains a significant challenge nowadays. It is a chronic neurological disease that can affect individuals of all ages. Moreover, aging can affect epilepsy detection significantly which makes epilepsy detection more challenging^6,7^. The etiology of epilepsy is heterogeneous, with variations observed among individuals. In certain instances, it is associated with underlying conditions such as traumatic brain injuries or developmental disorders^8^. Yet, the exact causes of epilepsy are still unknown and epilepsy detection presents a substantial challenge.

Recent advancements in diagnostic approaches allowed researchers and clinicians to shift from subjective assessments to more objective and quantifiable tests, recognized by improved accuracy since they provide more accurate results. Establishing a diagnosis of epilepsy most of the time involves a review of the exhaustive medical history and a physical examination, along with a series of tests. These tests include an electroencephalogram (EEG) to measure brain activity and imaging scans such as functional magnetic resonance imaging (fMRI) to identify any structural abnormalities in the brain. Epilepsy brings substantial changes to certain parts of the brain regions such as the hippocampus, amygdala, frontal cortex, temporal cortex, and olfactory cortex^9–11^. However, these structural changes occur in a rather slow progression.

EEG is a non-invasive test that measures and records the electrical activity of the brain using electrodes placed on the scalp^12,13^. During an EEG, the electrical signals generated by the brain are amplified and displayed as waveforms on a computer screen. The patterns observed in the EEG can provide valuable information including the presence of epilepsy, seizure classification, and seizure prediction^14^. Therefore, EEG plays a crucial role in the detection of epilepsy. Correspondingly, it will also facilitate the treatment of epilepsy which will be immensely helpful for human beings.

## Literature review

In a study, a wavelet scalogram was utilized in the dataset described by Andrzejak et al.^15^ with Alex net and achieved 100% accuracy^16^. Fourier-based synchro squeezing transform (SST) and convolutional neural network were applied in the publicly available CHB-MIT dataset and achieved 99.63% accuracy^17^. Multi-view feature learning was utilized with convolutional deep learning applied in the CHB-MIT dataset achieving 94.37% accuracy^18^. Till now different studies utilizing neural networks for epilepsy detection are discussed. Although, in various studies, researchers utilized neural networks but, it is having some dilemma too. Here, EEG signals are converted into images by different transform analysis which is an arduous and time-consuming task. Additionally, the convolutional neural network automatically extracts features from the image. Hence, researches are not aware of the features that play significant role in epilepsy detection. In a study, Discrete wavelet transform was used to decompose the data into sub-bands and then the wavelet energy distribution of each band was used as a feature set from the dataset described by Andrzejak et al.^15^ utilized in artificial neural network (ANN) for classification achieved 95.0% accuracy^19^. However, ANN is very complex and it requires copious data for training. Thus, it is very time-consuming. Several machine learning models were used for epilepsy detection and they exhibited promising results. One study utilized wavelet transform on a dataset described by Andrzejak et al.^15^with K means clustering has achieved 96.67% accuracy^20^. Another study used the optimum allocation technique on the dataset described by Andrzejak et al.^15^ with the logistic model tree achieved 96.67% accuracy^21^. Fuzzy distribution entropy (fDisEn) and wavelet packet decomposition were extracted from the dataset described by Andrzejak et al.^15^ and were utilized in K-nearest neighbor. This method achieved 98.33% accuracy as per reference^22^. Symlet wavelet processing and grid search optimizer featured extracted from dataset described by Andrzejak et al.^15^ and were used in gradient boosting machine. This method achieved 96.10% accuracy^1^. In some studies, researchers segmented the EEG signals. Thus, prolonged EEG signals were not required for classification. In a study, the 5 s epileptic segment was utilized with sample entropy and distributed entropy, using GA-SVM (genetic algorithm for feature selection and parameter optimization of support vector machine) on the dataset described by Andrzejak et al.^15^ and achieved a maximum AUC of 96.67%^23^. In practical while data is recorded from humans there are so many noises and artifacts which makes epilepsy detection more challenging. Spectrogram images of EEG signals were utilized in a pre-trained convolutional neural network applied in the TUH dataset, which achieved the highest 88.30% accuracy as per as the reference^24^. A one-dimensional deep convolutional neural network was used for the automated identification of abnormal EEG signals without any feature extraction and applied in the TUH dataset, which achieved 79.34% accuracy^25^.

## Research gap

A common practice in epilepsy research is often based on the use of smooth, noise-free datasets. As the data is mostly noise-free and smooth, researchers only utilized filters such as band pass, high pass, and low pass to put an end to noise from the signal. Therefore, researchers achieved significantly higher accuracy for epilepsy detection. In contrast, the real-world EEG signals are often contaminated by noise and various kinds of artifacts, which raises big questions regarding the practicality of the aforementioned methods. Therefore, the methodology proposed by the researchers will not work flawlessly.

Epilepsy detection from segmented EEG has supplementary utility. However, practical use of segmented EEG in detection still remains a limiting challenge for epilepsy diagnosis, since the accuracy rates drop much below certain conditions that remain to be fully understood. Therefore, although segmented has added value for epilepsy detection, researchers cannot take advantage of it.

Different researchers utilized non-identical features for their study^7,26–28^**.** Additionally, researchers claimed that the feature utilized in their study is the most prominent feature for epilepsy detection^26^. Thus, it is not transparent that actually which features play consequential role in epilepsy detection. Moreover, there appears to be no widely accepted significance order for epilepsy detection. Furthermore, researchers only have a few feature for their study^26,27^.

Researchers have not created any metric that can be utilized to exhibit the significance of the features for all the machine learning models. It is a notable issue since different features work differently with disparate machine learning models. Hence, it was not feasible for the researchers to construct a feature significance order for epilepsy detection that would remain accurate for all the models.

The field has thus far mostly focused on the use of either single machine learning or neural network-based models for epilepsy detection, leaving quite unexplored area of more promising hybrid models or the synergistic use of a few models. This narrow vision is missing the opportunity in which the fusion of different methodologies could make the system of epilepsy detection more accurate and robust. It points toward a very significant area for future research and development in this domain.

## Objective

The main problems that we are striving to address in this paper: are (1) achieving the utmost accuracy in epilepsy detection utilizing noisy data (TUH dataset) which are more likely to practical EEG signals. (2) Finding out the reason behind accuracy mitigation while utilizing segmented EEG signals. (3) Finding out the discriminant features and evolving the feature order for epilepsy detection. (4) proposing a new metric that can be utilized to exhibit the significance of the features for all the machine learning models. (5) proposing a new hybrid model that will detect epilepsy more precisely than a conventional single model. To the best of our knowledge, existing research has not specifically addressed these challenges and hence, our work represents a significant advance, offering innovative solutions rather than mere incremental progress. This highlights the potential for translational application and highlights the innovation and impact of our research. Therefore, this research outcome will be substantial enough for epilepsy detection.

## Methodology

### Data preparation and processing

In this study, we utilized the world’s largest publicly available EEG database which is called the TUH EEG corpus database^8^. The number of channels used for the recording of EEG signals is 32. Here, some channels are non EEG, such as (EEG-KKG, and EEG-RESP). We did not use these channels^29^. 10–20 electrode placement systems used in this dataset. This dataset carries annotation files by dint of it we can acquire knowledge about the event of each channel. Here, we used data from 10 disparate patients (5 epileptic, 5 normal). Delineate information regarding patients is provided in the supplementary information.

EEG may contain muscle activity, eye movement, power line interference, and interference from other devices. These are called noise of EEG signals. The primary purpose of EEG signals is to record the cerebral electrical activity of the brain. However, it may also record the electrical activity that is not generated from the cerebral region of the brain which is known as artifact of EEG signals. These noise and artifacts do not contain any significant information that may assist the analyze of EEG signals.

Noise and artifact evolve dilemma while analyzing EEG signals. Noise and artifact removal may increase the output of EEG signal analyze in a positive direction significantly. In the previous study, researchers utilized only filtering owing to remove the noise artifact. In this study, we have also utilized manual noise remove for better results.

### Signal analysis and feature extraction

A bandpass filter is the most effective way to remove noise from the EEG signal. In our study, EEG signals were passed across a bandpass filter with a cut-off frequency between 0.1 to 44 Hz, resulting in high-frequency noise along with low-frequency superfluous signal being detached. MATLAB EEGLAB was utilized for the visualization as well as filtering of the signal. EEG signals before as well as after filtering are depicted in supplementary information.

After filtering the signal, noise, and artifacts were obviated from the signal manually by taking advantage of the annotation files. To discern the noise, we employed the image exhibited in the annotation files. EEG signals before as well as after removing the noise along with the artifact are depicted in supplementary information.

As the TUH EEG corpus dataset comprises a multi-channel EEG signal, it is necessary to select the appropriate channels. We used statistical features such as mean, median, and standard deviation for channel selection. Initially, we calculated statistical features for each channel of the EEG signal. Subsequently, we constructed co-relation as well as reckoned the p-value of the mean, median, and standard deviation for each channel of the EEG signal. Afterward, we selected two channels that have the upmost co-relation and p-value.

In this study, we segmented the Normal EEG signal and Epileptic EEG signal into 5-s fragments. Then, this 5-s EEG signal was used for feature extraction. Different signal fragments have been used in other research studies such as 0.1 s^30^, 2 s^31,32^, 4 s^33^, 5 s^23,34^, and 60 s^25^.

In the context of signal processing, features refer to specific characteristics of a signal. Additionally, features provide more relevant information about the signal context. To extract features various mathematical computations as well as algorithms are applied to the raw signals. For instance, in the case of EEG signals, there are copious features. Different techniques are utilized to extract these features.

Based on the idea of signal processing, a signal can have many features. However, the significance of the features is not alike. Disparate features have different noteworthiness. Feature ranking depicts the importance of the features of that particular signal.

### Machine learning framework

A machine learning model is an algorithm that learns patterns from the given data to make prediction, and decisions without being explicitly programmed. These models initially process data, learn from data, and make decisions and predictions for unknown data.

Classification indices imply the performance of the machine learning models for classification tasks. These metrices indicate how well machine learning works, in making classification. Therefore, we can perceive that which model can be utilized for our stipulate classification task. In this study, we used four classification indices such as accuracy, precision, recall, f1 score.

Furthermore, to enhance the robustness of our findings, we used cross-validation. The basic idea of cross-validation is to divide the dataset into multiple subsets, train and test the model on different subsets, and then aggregate the results to get a more robust evaluation of the model's performance.

In this method, multiple base models are trained independently on the training data, each producing its predictions or decisions. The prime reason behind it is different models can capture different aspects of the data. All the models take the same feature value and make their predictions individually. Afterward, this prediction can be used for the classification or prediction that may outperform the single model.

A meta-classifier, is a higher-level model that combines the predictions or decisions of multiple base models to make a final prediction or classification. The purpose of the meta-classifier is to learn how to best combine the predictions of the base models to make a final prediction or classification. It takes into account the strengths and weaknesses of the base models and attempts to leverage the complementary information provided by them.

A key innovation in our methodology is the use of a stacking classifier which is the combination of base model and meta classifier. The idea behind stacking is to escalate predictive performance. In this study, we utilized two base model, and then the output of the base models were given to meta classifier.

### Proposed metrics

In this study, we proposed a metric for depicting the significance of features. Initially, all the feature ranks are calculated for different models using permutation feature importance^35–38^. Different models gave disparate feature ranks. To solve this dilemma, we proposed new metric called DF-A (discriminant feature–accuracy). In this metric, accuracy implies the cut-off accuracy for the selected model. For example, DF-90 implies the models that gave 90 percent or above accuracy are taken into account. Afterward, the cumulative order for selected models is calculated for all the features. Cumulative feature order means the summation of feature order for any particular feature for the selected models. The most discriminant feature has the least feature order value. Then, features are arranged in ascending order of the cumulative feature order. In this way, we calculated the value of DF-A that exhibits the importance of features of a particular signal for any particular task.

### Proposed model

**Figure 1:**
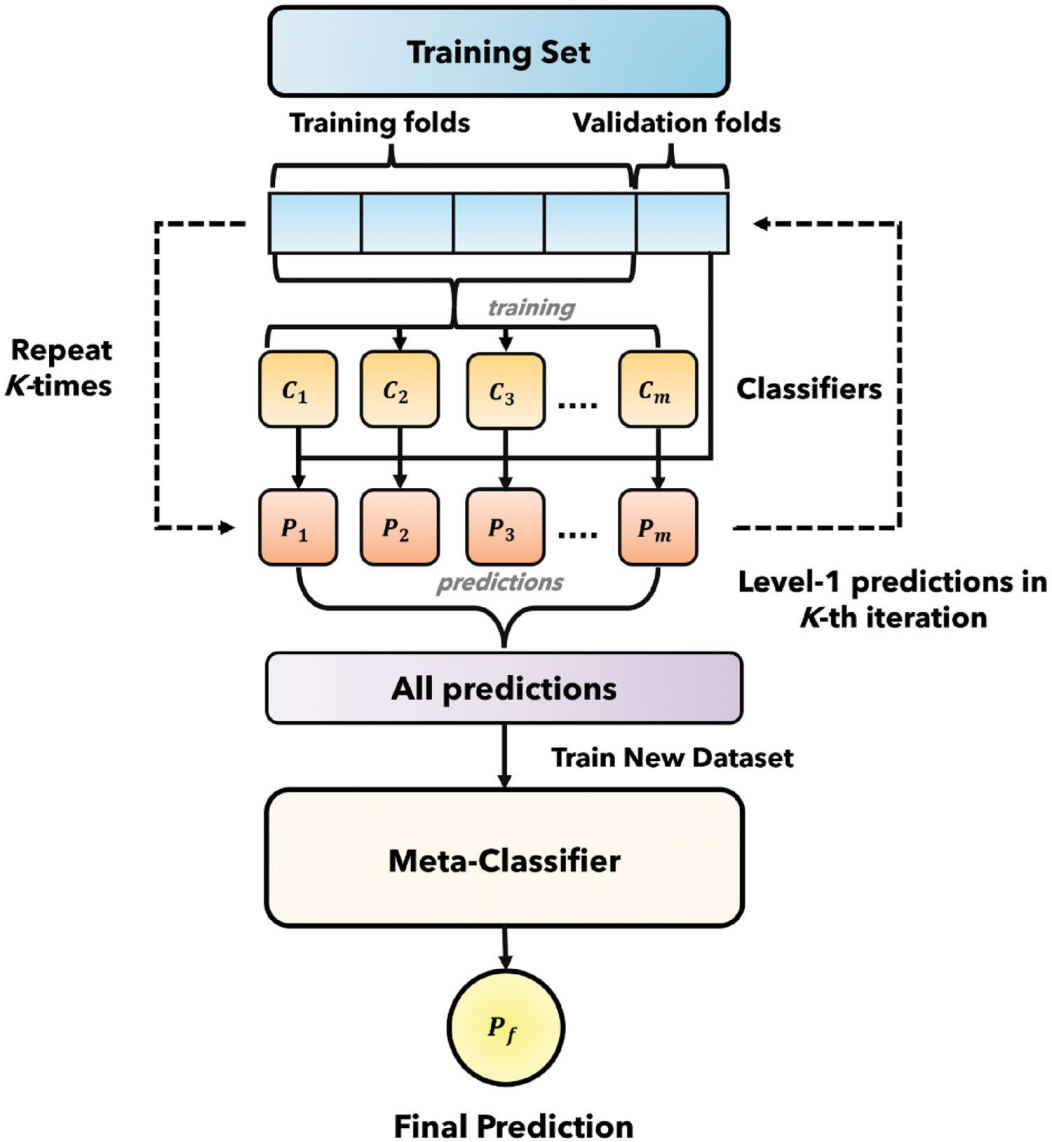
Stacking classifier (combining base model and meta classifier).

In this study, we utilized two models for ensemble to mitigate the computational complexity. The output prediction of the model is utilized to evolve a new dataset. Afterwards, this meta-classifier is used with the new dataset. In this way, our proposed model exhibited in Fig. 1 outperformed all other models.

### Proposed methodology

**Figure 2:**
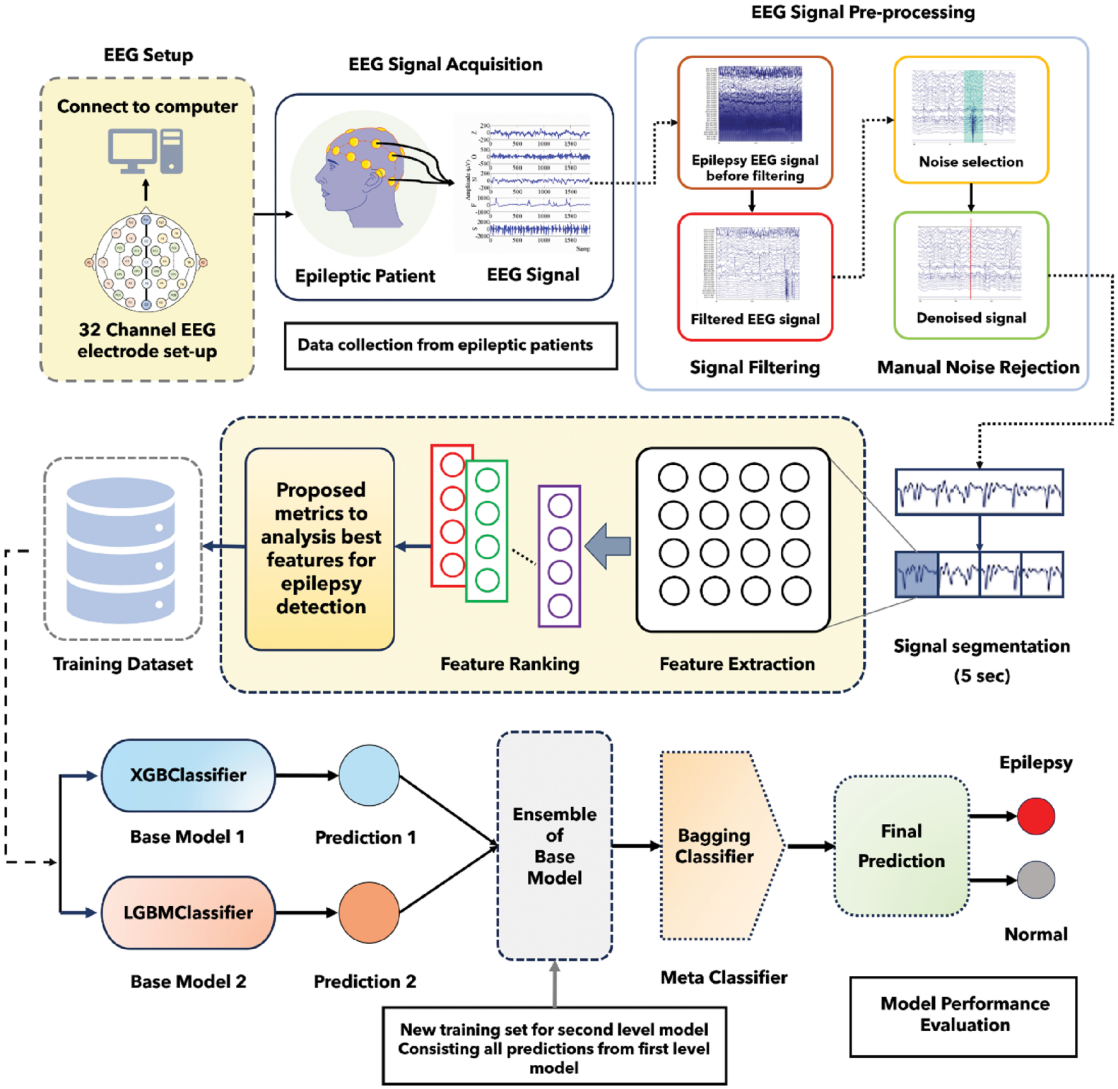
Graphical representation of the proposed methodology of this study.

Here, Fig. 2 depicts the novel methodology used in this study for epilepsy detection along with feature ranking.

## Result

### Single base model

The results for the single model with 32 feature datasets is depicted in supplementary information. We divided the classifier model into two parts such as right and left. In the left side, classifier models that exhibited accuracy 95% or above. On the other hand, Classifier models that exhibited lower than 95% accuracy are on the right side.

### DF-95 metrics

In order to exhibit the most significant feature DF-A metric was utilized. In this study, we selected 95% accuracy for DF-A metric as the accuracy parameter is variable and it can be altered. We used all the classifier models that exhibited 95% accuracy or above in single-model epilepsy detection task. Feature orders were calculated for all 32 features and seven classifier models that exhibited selected 95 accuracy or above. In Table 1, first column shows the 32 features used in this study. From column 2 to column 8 shows the feature order of 32 features used in this study for selected seven classifiers. Here, feature order was calculated by permutation feature importance. Afterward, in column 9 shows cumulative ranking order which is calculated by simple arithmetic addition. Subsequently, DF-95 (discriminant features–accuracy) is calculated by observing the cumulative ranking order. The feature achieving the least cumulative ranking order is entitled as the most discriminant feature. In this way, all 32 features are ranked for DF-95. The results are depicted in Table 1.

**Table 1:** Feature order, cumulative feature order and results for the DF-95.

	XGB classifier order	LGBM classifier order	RandomForest classifier order	ExtraTrees classifier order	Bagging classifier order	AdaBoost classifier order	Decision tree classifier order	Cumulative ranking order	DF-95
1.kurtosis	21	15	28	26	15	34	17	156	24
**2.mean absolute deviation**	**4**	**1**	**7**	**2**	**8**	**19**	**4**	**45**	**3**
3. mean	27	23	30	32	19	27	12	170	28
4.median	30	30	31	31	32	14	23	191	34
**5.root mean square**	**7**	**7**	**5**	**9**	**20**	**3**	**24**	**75**	**8**
6.skewness	13	17	26	19	16	13	8	112	14
7.standard deviation	11	27	13	6	7	12	22	98	12
8.variance	32	34	12	21	30	22	16	167	26
**9.correlation dimension**	**5**	**4**	**9**	**11**	**17**	**6**	**3**	**55**	**5**
**10.largest lyapunov exponent**	**16**	**3**	**11**	**1**	**18**	**1**	**7**	**57**	**6**
**11.peak max**	**9**	**5**	**20**	**7**	**29**	**5**	**11**	**86**	**10**
12.peak min	20	13	29	24	21	15	19	141	20
13.instantaneous frequency max	18	10	33	30	14	11	18	134	18
14.instantaneous frequency min	12	8	19	25	28	2	21	115	16
15.energy	34	33	17	27	10	28	29	178	30
16.power	31	32	8	16	27	30	32	176	29
17.approximate entropy	10	14	6	3	23	32	34	122	17
18.harmonic mean	26	28	34	34	5	33	31	191	33
19.mode	29	26	32	33	4	31	25	180	31
20.shannon entropy	17	21	21	22	34	16	30	161	25
**21.AR1**	**14**	**12**	**10**	**5**	**13**	**20**	**9**	**83**	**9**
22.AR2	3	20	2	8	26	21	10	90	11
23.AR3	25	18	24	28	3	23	26	147	19
**24.AR4**	**1**	**19**	**1**	**10**	**1**	**10**	**1**	**43**	**2**
**25.AR5**	**6**	**16**	**18**	**13**	**2**	**7**	**5**	**67**	**7**
26.AR6	15	11	16	23	11	17	14	107	13
27.AR7	23	9	14	18	6	24	20	114	15
28.AR8	24	25	22	20	12	18	27	148	21
29. activity	33	31	15	15	22	25	15	156	23
30.mobility	28	24	23	14	25	26	28	168	27
**31.complexity**	**8**	**2**	**4**	**4**	**24**	**4**	**6**	**52**	**4**
32.recurrence rate	22	22	27	29	31	9	13	153	22
**33.laminarity**	**2**	**6**	**3**	**12**	**9**	**8**	**2**	**42**	**1**
34.MLV	19	29	25	17	33	29	33	185	32

### Base 2 meta stacking classifier

To reduce the computational complexity only 2 models were used as a Base model. XGB Classifier along with LGBM Classifier as a base classifier performed better than any other model. It may happened since they also performed significantly better as a single model for epilepsy detection with a 32-feature dataset. Therefore, results are depicted while they were utilized as base classifiers and others models as meta classifiers. We also investigated the impact of cross-validation in this study and utilized disparate CV values such as 10, 25, 50, 75, and 100.

Supplemental information depicts the results of models for cross-validation value 10. We can perceived that the highest accuracy, precision, recall, and F1 score were respectively 0.95 (+ /− 0.04), 0.96 (+ /− 0.06), 1.00 (+ /− 0.00), and 0.95 (+ /− 0.04). However, some models exhibited 1.00 (+ /− 0.00) recall, but accuracy, precision, and F1 score were low for them.

Supplemental information depicts the results of models for cross-validation value 25. We can perceived that the highest accuracy, precision, recall, and F1 score were respectively 0.95 (+ /− 0.04), 0.96 (+ /− 0.06), **1.00 (+ /**− **0.00),** and 0.96 (+ /− 0.06). However, some models exhibited 1.00 (+ /− 0.00) recall, but accuracy, precision, and F1 score were low for them.

Supplemental information depicts the results of models for cross-validation value 50. we can perceived that the highest accuracy, precision, recall, and F1 score were respectively **0.97 (+ /**− **0.06)**, **0.97 (+ /**− **0.07)**, **0.98 (+ /**− **0.05),** and **0.97 (+ /**− **0.05)**. Although some models exhibited 1.00 (+ /− 0.00) recall, but accuracy, precision, and F1 score were low for them.

Table 2 depicts the results of models for cross-validation value 75. We can perceived that the highest accuracy, precision, recall, and F1 score were respectively **0.98 (+ /**− **0.05)**, **0.98 (+ /**− **0.07)**, **0.98 (+ /**− **0.05)** and **0.98 (+ /**− **0.04)**. Although some models exhibited 1.00 (+ /− 0.00) recall, but accuracy, precision, and F1 score were low for them. Meta–Bagging Classifier provided the upmost results when it was used as a meta-classifier.

**Table 2:** Results of model for CV 75.

Model	Accuracy	Precision	Recall	F1 score
Base-XGB Classifier	0.97 (+ /− 0.05)	0.98 (+ /− 0.07)	0.98 (+ /− 0.05)	0.97 (+ /− 0.05)
Base-LGBM Classifier	0.97 (+ /− 0.06)	0.97 (+ /− 0.07)	0.98 (+ /− 0.06)	0.97 (+ /− 0.05)
Meta-Random Forest Classifier	0.97 (+ /− 0.05)	0.98 (+ /− 0.07)	0.98 (+ /− 0.06)	0.97 (+ /− 0.05)
Meta-Extra Trees Classifier	0.97 (+ /− 0.05)	0.97 (+ /− 0.07)	0.98 (+ /− 0.06)	0.97 (+ /− 0.05)
**Meta**-**Bagging Classifier**	**0.98 (+ /**− **0.05)**	**0.98 (+ /**− **0.07)**	**0.98 (+ /**− **0.05)**	**0.98 (+ /**− **0.04)**
Meta-AdaBoost Classifier	0.97 (+ /− 0.06)	0.97 (+ /− 0.07)	0.98 (+ /− 0.06)	0.97 (+ /− 0.05)
Meta-Decision Tree Classifier	0.97 (+ /− 0.05)	0.97 (+ /− 0.07)	0.98 (+ /− 0.05)	0.98 (+ /− 0.05)
Meta-SVC	0.97 (+ /− 0.05)	0.97 (+ /− 0.07)	0.98 (+ /− 0.04)	0.97 (+ /− 0.05)
Meta-KNeighbors Classifier	0.97 (+ /− 0.05)	0.98 (+ /− 0.06)	0.97 (+ /− 0.06)	0.97 (+ /− 0.05)
Meta-Label Spreading	0.97 (+ /− 0.05)	0.97 (+ /− 0.07)	0.98 (+ /− 0.04)	0.97 (+ /− 0.05)
Meta-Label Propagation	0.97 (+ /− 0.05)	0.97 (+ /− 0.07)	0.98 (+ /− 0.04)	0.97 (+ /− 0.05)
Meta-Nu SVC	0.97 (+ /− 0.05)	0.97 (+ /− 0.07)	0.98 (+ /− 0.05)	0.97 (+ /− 0.05)
Meta-SGD Classifier	0.97 (+ /− 0.05)	0.98 (+ /− 0.06)	0.97 (+ /− 0.06)	0.97 (+ /− 0.05)
Meta-Linear SVC	0.97 (+ /− 0.05)	0.97 (+ /− 0.07)	0.98 (+ /− 0.04)	0.97 (+ /− 0.05)
Meta-Calibrated Classifier CV	0.97 (+ /− 0.05)	0.97 (+ /− 0.07)	0.98 (+ /− 0.04)	0.97 (+ /− 0.05)
Meta-Passive Aggressive Classifier	0.51 (+ /− 0.02)	0.51 (+ /− 0.02)	1.00 (+ /− 0.00)	0.67 (+ /− 0.02)
Meta-Ridge Classifier	0.97 (+ /− 0.05)	0.97 (+ /− 0.07)	0.98 (+ /− 0.04)	0.97 (+ /− 0.05)
Meta-Ridge Classifier CV	0.97 (+ /− 0.05)	0.97 (+ /− 0.07)	0.98 (+ /− 0.04)	0.97 (+ /− 0.05)
Meta-Perceptron	0.97 (+ /− 0.05)	0.98 (+ /− 0.06)	0.97 (+ /− 0.06)	0.97 (+ /− 0.05)
Meta-Quadratic Discriminant Analysis	0.49 (+ /− 0.02)	0.00 (+ /− 0.00)	0.00 (+ /− 0.00)	0.00 (+ /− 0.00)
Meta-Nearest Centroid	0.97 (+ /− 0.05)	0.98 (+ /− 0.06)	0.97 (+ /− 0.06)	0.97 (+ /− 0.05)
Meta-Bernoulli NB	0.97 (+ /− 0.05)	0.97 (+ /− 0.07)	0.98 (+ /− 0.04)	0.97 (+ /− 0.05)
Meta-Gaussian NB	0.97 (+ /− 0.05)	0.97 (+ /− 0.07)	0.98 (+ /− 0.04)	0.97 (+ /− 0.05)
Meta-Dummy Classifier	0.51 (+ /− 0.02)	0.51 (+ /− 0.02)	1.00 (+ /− 0.00)	0.67 (+ /− 0.02)

Supplemental information depicts results of models for cross-validation value 100. We can perceived that the highest accuracy, precision, recall, and F1 score were respectively 0.97 (+ /− 0.06), 0.97 (+ /− 0.07), 0.98 (+ /− 0.05), and 0.97 (+ /− 0.05). Although some models exhibited 1.00 (+ /− 0.00) recall, but accuracy, precision and F1 score were low for them.

## Discussion

### Findings of the study

The most significant finding of this study are proposed combination of the Base-2-Meta model that exhibits higher accuracy than the usually used single model. Additionally, finding out the most discriminant feature for epilepsy detection. Moreover, we have proposed a metric DF-A for depicting the most discriminant feature that is persistent with most of the models that give A accuracy or above. Furthermore, we have also investigated the influence of cross-validation in this study. In addition, one of the most significant findings of this study is to work with a challenging dataset containing copious noise and artifacts and achieving higher accuracy by manual noise rejection and filtering. Besides, achieving upmost accuracy with 5-s fragments of EEG signals.

In our proposed model, we utilized XGB and LGBM classifiers as base classifiers. We used the Bagging Classifier as a meta-classifier. We observed from the results that our proposed model gave better accuracy the single base model. Accuracy, precision, recall and F1 score for this Base-2-Meta model are respectively 0.98 (+ /− 0.05), 0.98 (+ /− 0.07), 0.98 (+ /− 0.05) and 0.98 (+ /− 0.04). Additionally, it also renders better results than other Base-2-Meta models. Initially, base model used the dataset and made the initial prediction. The probability of prediction is used as a dataset for the meta-classifier.

We observed from the study that some models gave better accuracy when they were used as meta classifiers than utilized as a single classifier model. We realized from this escalation of results that the output result substantially depends on the Base classifier. While the classifier model works as a single classifier model they used the main feature dataset. Conversely, when they worked as a meta classifier they used the dataset evolved from the probability of prediction predicted by the Base classifier.

Our proposed metric, DF-95 rendered the rankings of the features for epilepsy detection. Previously, there was confusion about the most discriminant feature since different model implies different features as the most discriminant. Additionally, different researchers also claim different features as the most discriminant. However, our proposed metric resolves this dilemma.

We investigated the impact of cross-validation in this study. We observed that when we increased the cross-validation value model training time increased significantly. However, we also observed the escalation of results with the cross-validation. Thus, we have found a tradeoff between execution time and accuracy for cross-validation. Although a higher cross-validation value increases the complexity, it gives better results.

EEG signals contain various noise and artifacts and they lower the results of detection. However, researchers in their research work mostly utilized noise-free and smooth datasets. Thus, they were able to achieve the upmost accuracy with their proposed methodology. However, with the practical data their proposed models do not work up to the mark.

In this study, we utilized a manual noise rejection technique along with filtering the data. In addition, we utilized a novel statistical channel selection procedure to find out the most significant EEG channel. Afterward, features were extracted from these channels. Therefore, our proposed methodology achieved significant accuracy even with the dataset containing noise and artifacts.

We utilized fragments of EEG signals. Thus, a prolonged EEG signal is not required for epilepsy detection. In cases where only a small fragment of EEG signals is present, our proposed methodology can be utilized to accurately detect epilepsy with those small EEG signals.

We have found a tradeoff between signal-to-noise ratio (SNR) with the length of the EEG signals. When the length of the EEG signal is reduced, detection accuracy is also mitigated. Researchers utilized only filtering for noise eradication. However, filtering alone cannot eradicate the noise and artifact accurately. Therefore, when the length of the signals is reduced the signal part of the signal is reduced considerably but noise and artifacts do not reduce at that high level. Thus, a significant reduction of results is observed while the length of the signal is reduced.

Conversely, our methodology worked significantly even with the 5 s fragment of the EEG signals. In our proposed method, we utilized novel manual noise and artifact rejection techniques along with filtering. In this way, noise and artifacts can be removed significantly better than only filtering. Therefore, while the error rate with signal fragments for other studies is significantly high, in our study error rate with 5 s fragments of data is only 2%.

### Comparison

We have achieved up-to-the-mark results with our proposed methodology even though we utilized a dataset that contains copious noise and artifact-like practical data. We achieved this sublime result owing to our novel approaches. Therefore, our proposed methodology will be very useful for the practical application. Tables 3 and 4 exhibit the comparison of our study with previous studies conducted on the same dataset. From the comparison, it is evident that our study surpasses previous studies. This superiority is attributed to our innovative approach.

**Table 3:** Performance comparison of the proposed method with other methods using three parameters (Epoch Size, Results, and Classifier).

Classifier	Epoch Size	Results	Reference
One-dimensional deep convolutional neural network	60 Sec	Error rate 20.6%	^25^
Base-2-Meta	5 Sec	Error rate 2%	This work

**Table 4:** Comparison results of the proposed method with previous works.

Feature	Classifier	Dataset	Result	Reference
Spectrogram	Convolutional neural network	TUH	Acc. 88.30%	^39^
Automated identification without feature extraction	One-dimensional deep convolutional neural network	TUH	Acc. 79.34%	^25^
32 Features extracted from EEG signals	Base-2-Meta	TUH	Acc. 98%	This work

### Limitations

In our study, although we achieved 98% accuracy, it can be further improved with additional novel techniques in the future. Although, we utilized 32 features, the number of features can be further improved which might raise the result. we rendered a ranking of features for epilepsy detection. However, this order of features was only calculated between the 32 features utilized in this study. In the future, if further features are augmented then the orders of the features can be altered. The result of our proposed metric also varied with the accuracy parameter added with it. We utilized 5 s fragments of EEG data. Therefore, while EEG data is less than 5 s, our proposed methodology cannot detect epilepsy as accurately as the result exhibited in Table 3 and Table 4. Although our proposed methodology has a few drawbacks, it provides intriguing insights for epilepsy detection. However, in the future we are looking forward to providing more significant insights for epilepsy detection and resolving these drawbacks.

## Conclusion

Although epilepsy detection is a challenging task, our proposed methodology can do it merely accurately. We utilized a novel manual noise rejection technique with filtering and extracted copious features for epilepsy detection. Afterward, a novel Base-2-Meta stacking model is utilized for the detection of epilepsy. Even though with the noisy data our proposed method can work up to the mark. We have also discovered the most discriminant features order in this study. In addition, our proposed metric DF-A used to exhibit the most discriminant feature unprecedentedly. We have also achieved significantly higher accuracy with 5 s fragments of EEG signals. The influence of cross-validation for the detection of epilepsy is investigated in this study. Our investigation provided significant insights for epilepsy detection. These insights can be considerably useful for the detection and treatment of epilepsy.

### Supplementary Information

Supplementary Information.

## Data Availability

The TUH EEG Epilepsy Corpus dataset was collected from the web link- https://isip.piconepress.com/projects/tuh_eeg/html/downloads.shtml.
