# Supplementary material for: Performance investigation of epilepsy detection from noisy EEG signals using base-2-meta stacking classifier
Source: Sci Rep. 2024 May 11;14:10792. doi: 10.1038/s41598-024-61338-2 (PMC11088643; doi:10.1038/s41598-024-61338-2)
Supplement: Supplementary file 1 — Supplementary Information. [file 41598_2024_61338_MOESM1_ESM.docx]

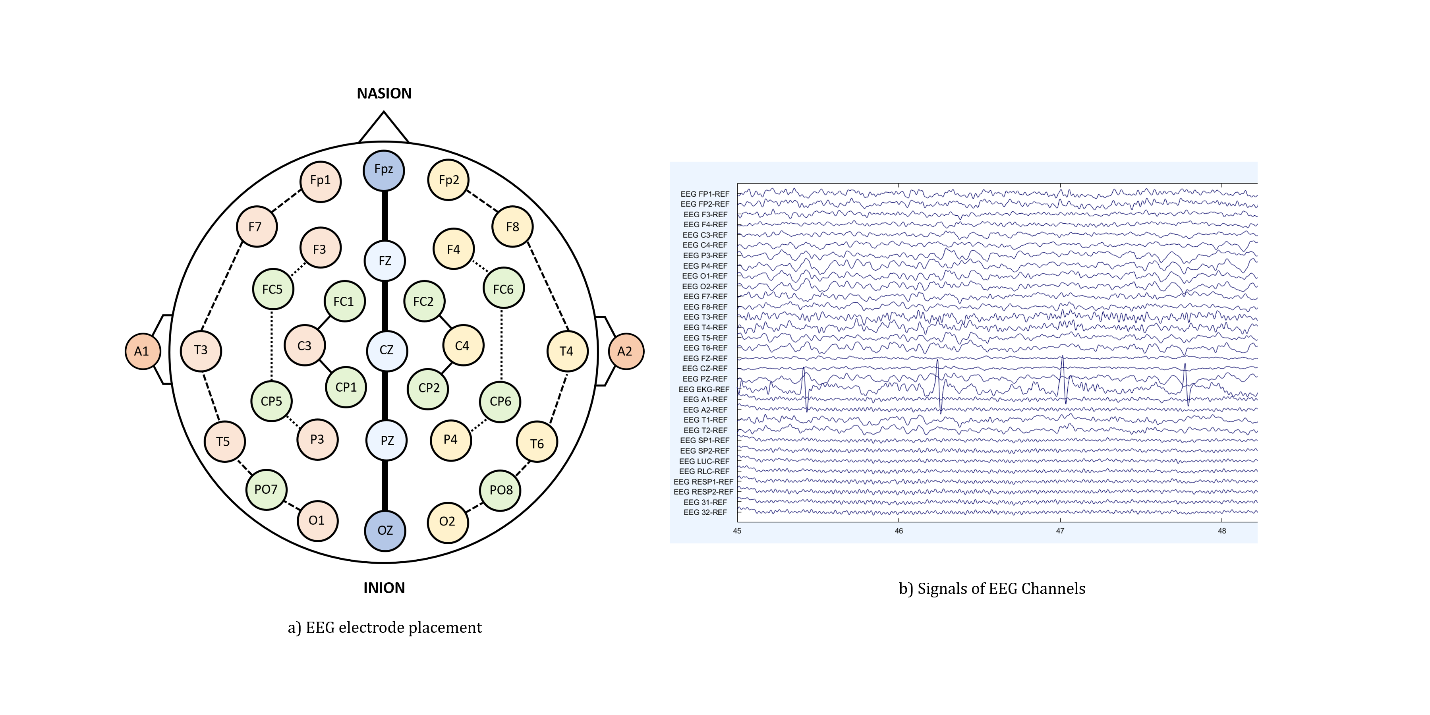
 Figure 1: Graphical representation of EEG electrode placement and corresponding EEG signal. REF is referred to as a reference electrode (an electrode connected to the left ear) according to the electrode file of this dataset.


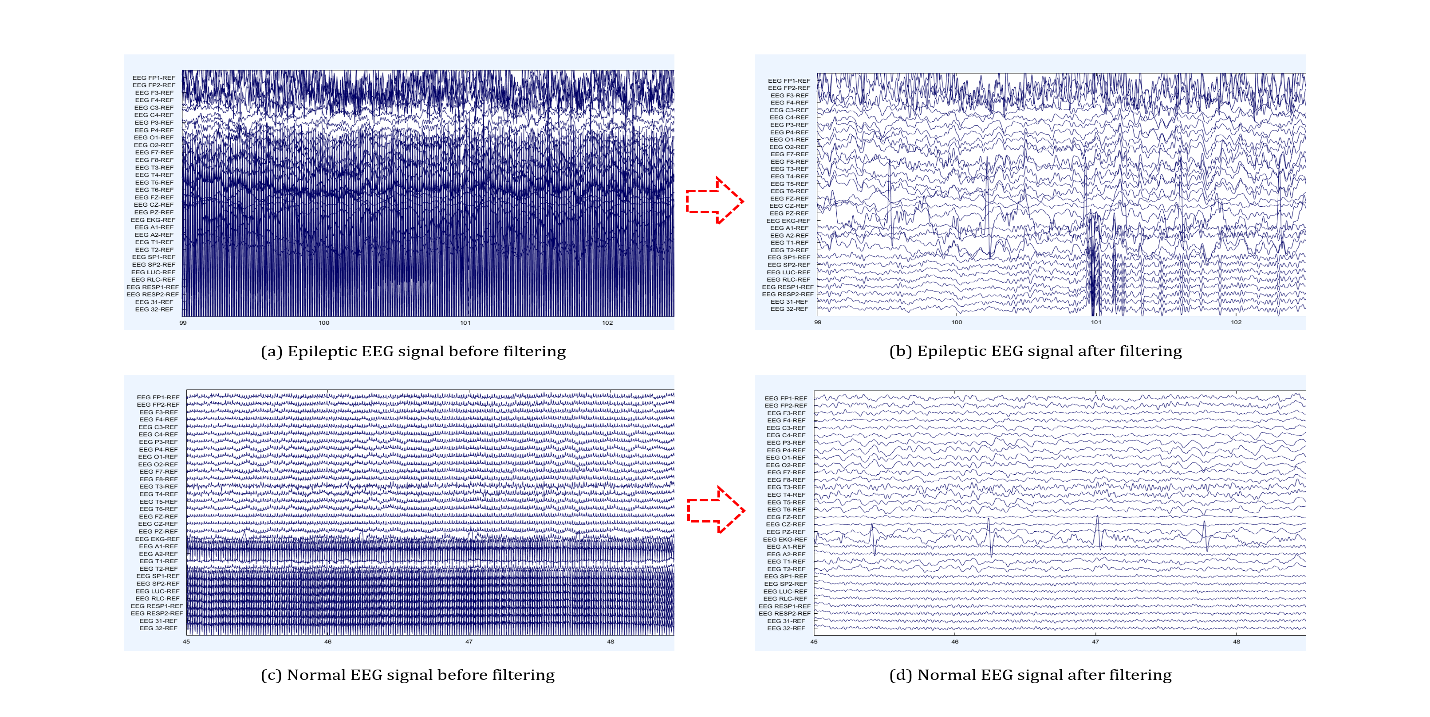
 Figure 2: EEG signal (Epilepsy and Normal) before and after filtering


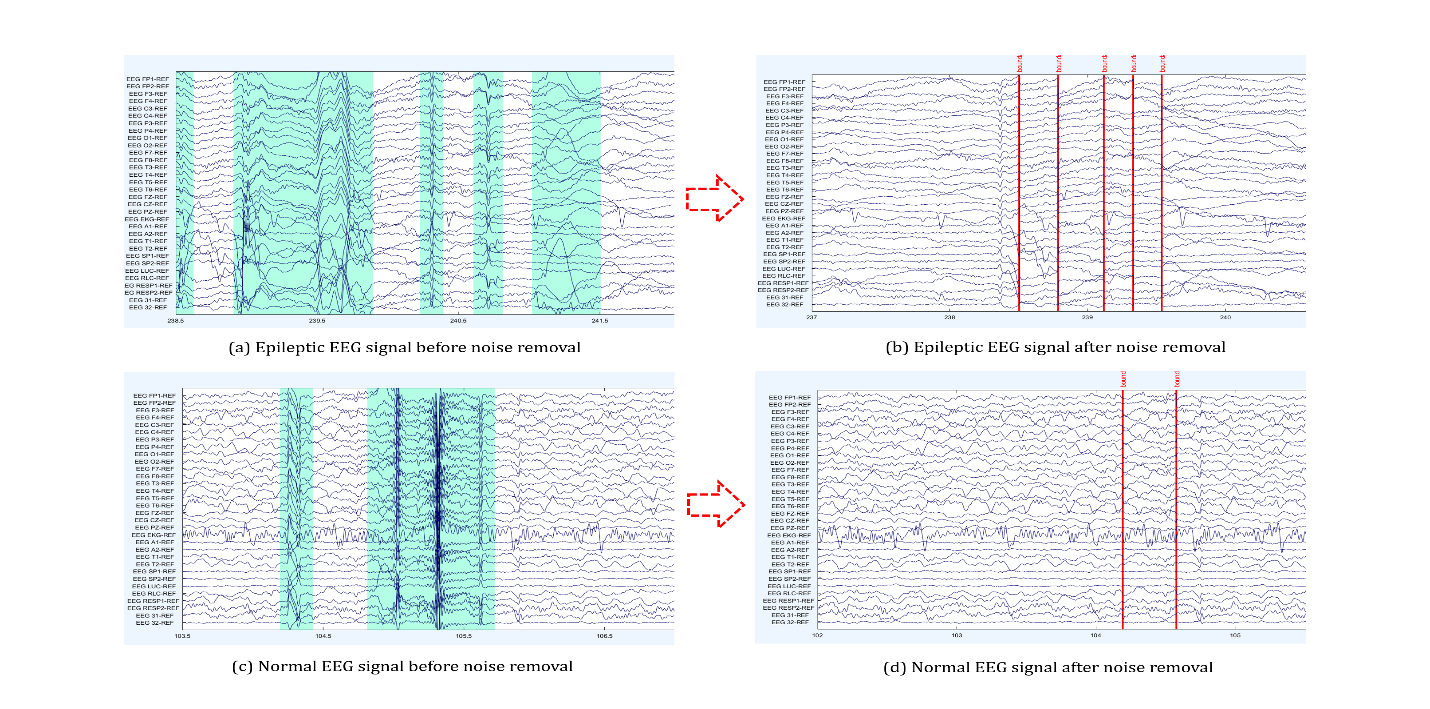
 Figure 3: EEG signal (Epilepsy and Normal) before and after manual noise rejection

Table 1. Patients Information

| **Types** | **Patient Id** | **Medications** | **Sex** | **Age** | **Heart Rate** | **Selected channels** |
| --- | --- | --- | --- | --- | --- | --- |
| **Epilepsy** | 258 | Dilantin, Phenobarbital, and Baclofen | M | 41 | 84 | 8(P4-REF),  16(T6-REF) |
|  | 675 | Albuterol | F | 4 | 126 | 3(F3-REF), 23(T1-REF) |
|  | 1027 | Dilantin and Phenobarbital | M | 50 | 72 | 1(FP1), 11(F7) |
|  | 1770 | Trileptal, Valproate, and Klonopin | F | 26 | 126 | 2(FP2-REF), 26(SP2-REF) |
|  | 1984 | Trileptal | M | 6 | 72 | 3(F3-REF), 23(T1-REF) |
| **Normal** | 258 | Dilantin, Phenobarbital, and Baclofen | M | 41 | 84 | 2(FP2-REF), 16(T6-REF) |
|  | 629 | Tegretol and Topamax | F | 22 | 78 | 9(O1-REF), 16(T6-REF) |
|  | 1027 | Dilantin and Phenobarbital | M | 50 | 72 | 1(FP1-REF), 11(F7-REF) |
|  | 1278 | Dilantin, Topamax, Remeron, and Reglan | F | 33 | 96 | 1(FP1-REF), 2(FP2-REF) |
|  | 1981 | Tegretol | M | 56 | 72 | 2(FP2-REF), 24(T2-REF) |

Table 2. Extracted feature from EEG signals

| 1.kurtosis | 10.Largest lyapunov exponent | 19.mode | 28.AR8 |
| --- | --- | --- | --- |
| 2.mean absolute deviation | 11.Peak Max | 20.Shannon_entropy | 29.Activity |
| 3.mean | 12.Peak Min | 21.AR1 | 30.Mobility |
| 4.median | 13.Instantaneous Frequency Max | 22.AR2 | 31.Complexity |
| 5.root mean square | 14.Instantaneous Frequency Min | 23.AR3 | 32.Recurrence_rate |
| 6.skewness | 15.Energy | 24.AR4 | 33.Laminarity |
| 7.standard deviation | 16.Power | 25.AR5 | 34.MLV |
| 8.variance | 17.Approximate_entropy | 26.AR6 |  |
| 9.Correlation Dimension | 18.Harmonic_mean | 27.AR7 |  |

Table 3. Results of accuracy for single Base model

| **Accuracy > 0.95** | | | |
| --- | --- | --- | --- |
| **Model** | | **Accuracy** | |
| XGB Classifier | | 0.97 | |
| LGBM Classifier | | 0.97 | |
| Random Forest Classifier | | 0.97 | |
| Extra Trees Classifier | | 0.97 | |
| Bagging Classifier | | 0.97 | |
| AdaBoost Classifier | | 0.96 | |
| Decision Tree Classifier | | 0.95 | |
| **Accuracy < 0.95** | | | |
| **Model** | **Accuracy** | **Model** | **Accuracy** |
| SVC | 0.9 | Logistic Regression | 0.85 |
| KNeighbors Classifier | 0.9 | Ridge Classifier | 0.84 |
| Label Spreading | 0.9 | Ridge Classifier CV | 0.84 |
| Label Propagation | 0.9 | Perceptron | 0.82 |
| Nu SVC | 0.89 | Quadratic Discriminant Analysis | 0.71 |
| SGD Classifier | 0.89 | Nearest Centroid | 0.68 |
| Linear SVC | 0.88 | Bernoulli NB | 0.68 |
| Calibrated Classifier CV | 0.88 | Gaussian NB | 0.67 |
| Linear Discriminant Analysis | 0.85 | Dummy Classifier | 0.48 |
| Passive Aggressive Classifier | 0.85 |  |  |

Table 4. results of model for CV 10

| Model | Accuracy | Precision | Recall | F1 score |
| --- | --- | --- | --- | --- |
| Base - XGB Classifier | 0.94 (+/- 0.04) | 0.95 (+/- 0.07) | 0.94 (+/- 0.06) | 0.94 (+/- 0.04) |
| Base - LGBM Classifier | 0.94 (+/- 0.05) | 0.95 (+/- 0.06) | 0.94 (+/- 0.08) | 0.94 (+/- 0.05) |
| Meta - Random Forest Classifier | 0.94 (+/- 0.05) | 0.95 (+/- 0.06) | 0.94 (+/- 0.08) | 0.94 (+/- 0.05) |
| Meta - Extra Trees Classifier | 0.95 (+/- 0.04) | 0.96 (+/- 0.06) | 0.94 (+/- 0.06) | 0.95 (+/- 0.04) |
| Meta - Bagging Classifier | 0.95 (+/- 0.04) | 0.96 (+/- 0.07) | 0.95 (+/- 0.06) | 0.95 (+/- 0.04) |
| Meta - AdaBoost Classifier | 0.94 (+/- 0.05) | 0.95 (+/- 0.06) | 0.94 (+/- 0.08) | 0.94 (+/- 0.05) |
| Meta - Decision Tree Classifier | 0.94 (+/- 0.05) | 0.95 (+/- 0.06) | 0.93 (+/- 0.08) | 0.94 (+/- 0.05) |
| Meta - SVC | 0.95 (+/- 0.04) | 0.95 (+/- 0.07) | 0.96 (+/- 0.05) | 0.95 (+/- 0.04) |
| Meta - KNeighbors Classifier | 0.94 (+/- 0.05) | 0.96 (+/- 0.06) | 0.92 (+/- 0.09) | 0.94 (+/- 0.05) |
| Meta - Label Spreading | 0.95 (+/- 0.04) | 0.95 (+/- 0.07) | 0.96 (+/- 0.05) | 0.95 (+/- 0.04) |
| Meta - Label Propagation | 0.95 (+/- 0.04) | 0.95 (+/- 0.07) | 0.96 (+/- 0.05) | 0.95 (+/- 0.04) |
| Meta - Nu SVC | 0.94 (+/- 0.05) | 0.96 (+/- 0.06) | 0.92 (+/- 0.09) | 0.94 (+/- 0.05) |
| Meta - SGD Classifier | 0.94 (+/- 0.05) | 0.96 (+/- 0.06) | 0.92 (+/- 0.09) | 0.94 (+/- 0.05) |
| Meta - Linear SVC | 0.95 (+/- 0.04) | 0.95 (+/- 0.07) | 0.96 (+/- 0.05) | 0.95 (+/- 0.04) |
| Meta - Calibrated Classifier CV | 0.95 (+/- 0.04) | 0.95 (+/- 0.07) | 0.96 (+/- 0.05) | 0.95 (+/- 0.04) |
| Meta - Passive Aggressive Classifier | 0.51 (+/- 0.00) | 0.51 (+/- 0.00) | 1.00 (+/- 0.00) | 0.67 (+/- 0.00) |
| Meta - Ridge Classifier | 0.95 (+/- 0.04) | 0.95 (+/- 0.07) | 0.96 (+/- 0.05) | 0.95 (+/- 0.04) |
| Meta - Ridge Classifier CV | 0.95 (+/- 0.04) | 0.95 (+/- 0.07) | 0.96 (+/- 0.05) | 0.95 (+/- 0.04) |
| Meta - Perceptron | 0.94 (+/- 0.05) | 0.96 (+/- 0.06) | 0.92 (+/- 0.09) | 0.94 (+/- 0.05) |
| Meta - Nearest Centroid | 0.94 (+/- 0.05) | 0.96 (+/- 0.06) | 0.92 (+/- 0.09) | 0.94 (+/- 0.05) |
| Meta - Bernoulli NB | 0.95 (+/- 0.04) | 0.95 (+/- 0.07) | 0.96 (+/- 0.05) | 0.95 (+/- 0.04) |
| Meta - Gaussian NB | 0.95 (+/- 0.04) | 0.95 (+/- 0.07) | 0.96 (+/- 0.05) | 0.95 (+/- 0.04) |
| Meta - Dummy Classifier | 0.51 (+/- 0.00) | 0.51 (+/- 0.00) | 1.00 (+/- 0.00) | 0.67 (+/- 0.00) |

Table 5. results of model for CV 25

| Model | Accuracy | Precision | Recall | F1 score |
| --- | --- | --- | --- | --- |
| Base - XGB Classifier | 0.96 (+/- 0.06) | 0.96 (+/- 0.08) | 0.96 (+/- 0.06) | 0.96 (+/- 0.06) |
| Base - LGBM Classifier | 0.96 (+/- 0.07) | 0.96 (+/- 0.09) | 0.97 (+/- 0.06) | 0.96 (+/- 0.06) |
| Meta - Random Forest Classifier | 0.96 (+/- 0.06) | 0.96 (+/- 0.08) | 0.97 (+/- 0.05) | 0.96 (+/- 0.05) |
| Meta - Extra Trees Classifier | 0.95 (+/- 0.07) | 0.96 (+/- 0.08) | 0.96 (+/- 0.07) | 0.96 (+/- 0.06) |
| Meta - Bagging Classifier | 0.96 (+/- 0.06) | 0.96 (+/- 0.08) | 0.97 (+/- 0.06) | 0.96 (+/- 0.06) |
| Meta - AdaBoost Classifier | 0.95 (+/- 0.07) | 0.95 (+/- 0.09) | 0.96 (+/- 0.07) | 0.96 (+/- 0.06) |
| Meta - Decision Tree Classifier | 0.96 (+/- 0.06) | 0.96 (+/- 0.09) | 0.97 (+/- 0.04) | 0.96 (+/- 0.06) |
| Meta - SVC | 0.96 (+/- 0.06) | 0.95 (+/- 0.09) | 0.98 (+/- 0.04) | 0.96 (+/- 0.06) |
| Meta - KNeighbors Classifier | 0.95 (+/- 0.07) | 0.96 (+/- 0.08) | 0.95 (+/- 0.08) | 0.95 (+/- 0.06) |
| Meta - Label Spreading | 0.96 (+/- 0.06) | 0.95 (+/- 0.09) | 0.98 (+/- 0.04) | 0.96 (+/- 0.06) |
| Meta - Label Propagation | 0.96 (+/- 0.06) | 0.95 (+/- 0.09) | 0.98 (+/- 0.04) | 0.96 (+/- 0.06) |
| Meta - Nu SVC | 0.96 (+/- 0.07) | 0.96 (+/- 0.08) | 0.97 (+/- 0.06) | 0.96 (+/- 0.06) |
| Meta - SGD Classifier | 0.95 (+/- 0.07) | 0.96 (+/- 0.08) | 0.95 (+/- 0.08) | 0.95 (+/- 0.06) |
| Meta - Linear SVC | 0.96 (+/- 0.06) | 0.95 (+/- 0.09) | 0.98 (+/- 0.04) | 0.96 (+/- 0.06) |
| Meta - Calibrated Classifier CV | 0.96 (+/- 0.06) | 0.95 (+/- 0.09) | 0.98 (+/- 0.04) | 0.96 (+/- 0.06) |
| Meta - Passive Aggressive Classifier | 0.51 (+/- 0.01) | 0.51 (+/- 0.01) | **1.00 (+/- 0.00)** | 0.67 (+/- 0.01) |
| Meta - Ridge Classifier | 0.96 (+/- 0.06) | 0.95 (+/- 0.09) | 0.98 (+/- 0.04) | 0.96 (+/- 0.06) |
| Meta - Ridge Classifier CV | 0.96 (+/- 0.06) | 0.95 (+/- 0.09) | 0.98 (+/- 0.04) | 0.96 (+/- 0.06) |
| Meta - Perceptron | 0.95 (+/- 0.07) | 0.96 (+/- 0.08) | 0.95 (+/- 0.08) | 0.95 (+/- 0.06) |
| Meta - Quadratic Discriminant Analysis | 0.49 (+/- 0.01) | 0.00 (+/- 0.00) | 0.00 (+/- 0.00) | 0.00 (+/- 0.00) |
| Meta - Nearest Centroid | 0.95 (+/- 0.07) | 0.96 (+/- 0.08) | 0.95 (+/- 0.08) | 0.95 (+/- 0.06) |
| Meta - Bernoulli NB | 0.96 (+/- 0.06) | 0.95 (+/- 0.09) | 0.98 (+/- 0.04) | 0.96 (+/- 0.06) |
| Meta - Gaussian NB | 0.96 (+/- 0.06) | 0.95 (+/- 0.09) | 0.98 (+/- 0.04) | 0.96 (+/- 0.06) |
| Meta - Dummy Classifier | 0.51 (+/- 0.01) | 0.51 (+/- 0.01) | 1.00 (+/- 0.00) | 0.67 (+/- 0.01) |

Table 6. results of model for CV 50

| Model | Accuracy | Precision | Recall | F1 score |
| --- | --- | --- | --- | --- |
| Base - XGB Classifier | 0.97 (+/- 0.06) | 0.97 (+/- 0.08) | 0.97 (+/- 0.05) | 0.97 (+/- 0.05) |
| Base - LGBM Classifier | 0.96 (+/- 0.06) | 0.96 (+/- 0.08) | 0.98 (+/- 0.05) | 0.97 (+/- 0.06) |
| Meta - Random Forest Classifier | 0.97 (+/- 0.06) | 0.96 (+/- 0.08) | 0.98 (+/- 0.05) | 0.97 (+/- 0.05) |
| Meta - Extra Trees Classifier | 0.96 (+/- 0.06) | 0.97 (+/- 0.08) | 0.97 (+/- 0.06) | 0.97 (+/- 0.05) |
| Meta - Bagging Classifier | **0.97 (+/- 0.06)** | **0.97 (+/- 0.07)** | **0.98 (+/- 0.05)** | **0.97 (+/- 0.05)** |
| Meta - AdaBoost Classifier | 0.96 (+/- 0.06) | 0.96 (+/- 0.08) | 0.97 (+/- 0.05) | 0.97 (+/- 0.06) |
| Meta - Decision Tree Classifier | 0.96 (+/- 0.06) | 0.96 (+/- 0.08) | 0.97 (+/- 0.05) | 0.97 (+/- 0.06) |
| Meta - SVC | 0.96 (+/- 0.07) | 0.96 (+/- 0.08) | 0.98 (+/- 0.05) | 0.97 (+/- 0.06) |
| Meta - KNeighbors Classifier | 0.97 (+/- 0.06) | 0.97 (+/- 0.07) | 0.97 (+/- 0.06) | 0.97 (+/- 0.05) |
| Meta - Label Spreading | 0.96 (+/- 0.07) | 0.96 (+/- 0.08) | 0.98 (+/- 0.05) | 0.97 (+/- 0.06) |
| Meta - Label Propagation | 0.96 (+/- 0.07) | 0.96 (+/- 0.08) | 0.98 (+/- 0.05) | 0.97 (+/- 0.06) |
| Meta - Nu SVC | 0.97 (+/- 0.07) | 0.96 (+/- 0.08) | 0.98 (+/- 0.05) | 0.97 (+/- 0.06) |
| Meta - SGD Classifier | 0.97 (+/- 0.06) | 0.97 (+/- 0.07) | 0.97 (+/- 0.06) | 0.97 (+/- 0.05) |
| Meta - Linear SVC | 0.96 (+/- 0.07) | 0.96 (+/- 0.08) | 0.98 (+/- 0.05) | 0.97 (+/- 0.06) |
| Meta - Calibrated Classifier CV | 0.96 (+/- 0.07) | 0.96 (+/- 0.08) | 0.98 (+/- 0.05) | 0.97 (+/- 0.06) |
| Meta - Passive Aggressive Classifier | 0.51 (+/- 0.01) | 0.51 (+/- 0.01) | 1.00 (+/- 0.00) | 0.67 (+/- 0.01) |
| Meta - Ridge Classifier | 0.96 (+/- 0.07) | 0.96 (+/- 0.08) | 0.98 (+/- 0.05) | 0.97 (+/- 0.06) |
| Meta - Ridge Classifier CV | 0.96 (+/- 0.07) | 0.96 (+/- 0.08) | 0.98 (+/- 0.05) | 0.97 (+/- 0.06) |
| Meta - Perceptron | 0.97 (+/- 0.06) | 0.97 (+/- 0.07) | 0.97 (+/- 0.06) | 0.97 (+/- 0.05) |
| Meta - Quadratic Discriminant Analysis | 0.49 (+/- 0.01) | 0.00 (+/- 0.00) | 0.00 (+/- 0.00) | 0.00 (+/- 0.00) |
| Meta - Nearest Centroid | 0.97 (+/- 0.06) | 0.97 (+/- 0.07) | 0.97 (+/- 0.06) | 0.97 (+/- 0.05) |
| Meta - Bernoulli NB | 0.96 (+/- 0.07) | 0.96 (+/- 0.08) | 0.98 (+/- 0.05) | 0.97 (+/- 0.06) |
| Meta - Gaussian NB | 0.96 (+/- 0.07) | 0.96 (+/- 0.08) | 0.98 (+/- 0.05) | 0.97 (+/- 0.06) |
| Meta - Dummy Classifier | 0.51 (+/- 0.01) | 0.51 (+/- 0.01) | 1.00 (+/- 0.00) | 0.67 (+/- 0.01) |

Table 7. results of model for CV 100

| Model | Accuracy | Precision | Recall | F1 score |
| --- | --- | --- | --- | --- |
| Base - XGB Classifier | 0.97 (+/- 0.06) | 0.97 (+/- 0.07) | 0.97 (+/- 0.06) | 0.97 (+/- 0.06) |
| Base - LGBM Classifier | 0.97 (+/- 0.06) | 0.97 (+/- 0.08) | 0.98 (+/- 0.06) | 0.97 (+/- 0.06) |
| Meta - Random Forest Classifier | 0.97 (+/- 0.06) | 0.97 (+/- 0.08) | 0.98 (+/- 0.07) | 0.97 (+/- 0.06) |
| Meta - Extra Trees Classifier | 0.97 (+/- 0.06) | 0.97 (+/- 0.07) | 0.98 (+/- 0.07) | 0.97 (+/- 0.06) |
| Meta - Bagging Classifier | 0.97 (+/- 0.06) | 0.97 (+/- 0.07) | 0.98 (+/- 0.05) | 0.97 (+/- 0.05) |
| Meta - AdaBoost Classifier | 0.97 (+/- 0.06) | 0.97 (+/- 0.07) | 0.98 (+/- 0.07) | 0.97 (+/- 0.06) |
| Meta - Decision Tree Classifier | 0.97 (+/- 0.06) | 0.97 (+/- 0.08) | 0.98 (+/- 0.06) | 0.97 (+/- 0.06) |
| Meta - SVC | 0.97 (+/- 0.06) | 0.97 (+/- 0.08) | 0.98 (+/- 0.05) | 0.97 (+/- 0.06) |
| Meta - KNeighbors Classifier | 0.97 (+/- 0.06) | 0.98 (+/- 0.07) | 0.97 (+/- 0.07) | 0.97 (+/- 0.06) |
| Meta - Label Spreading | 0.97 (+/- 0.06) | 0.97 (+/- 0.08) | 0.98 (+/- 0.05) | 0.97 (+/- 0.06) |
| Meta - Label Propagation | 0.97 (+/- 0.06) | 0.97 (+/- 0.08) | 0.98 (+/- 0.05) | 0.97 (+/- 0.06) |
| Meta - Nu SVC | 0.97 (+/- 0.07) | 0.97 (+/- 0.08) | 0.98 (+/- 0.05) | 0.97 (+/- 0.06) |
| Meta - SGD Classifier | 0.97 (+/- 0.06) | 0.98 (+/- 0.07) | 0.97 (+/- 0.07) | 0.97 (+/- 0.06) |
| Meta - Linear SVC | 0.97 (+/- 0.06) | 0.97 (+/- 0.08) | 0.98 (+/- 0.05) | 0.97 (+/- 0.06) |
| Meta - Calibrated Classifier CV | 0.97 (+/- 0.06) | 0.97 (+/- 0.08) | 0.98 (+/- 0.05) | 0.97 (+/- 0.06) |
| Meta - Passive Aggressive Classifier | 0.51 (+/- 0.02) | 0.51 (+/- 0.02) | 1.00 (+/- 0.00) | 0.67 (+/- 0.02) |
| Meta - Ridge Classifier | 0.97 (+/- 0.06) | 0.97 (+/- 0.08) | 0.98 (+/- 0.05) | 0.97 (+/- 0.06) |
| Meta - Ridge Classifier CV | 0.97 (+/- 0.06) | 0.97 (+/- 0.08) | 0.98 (+/- 0.05) | 0.97 (+/- 0.06) |
| Meta - Perceptron | 0.97 (+/- 0.06) | 0.98 (+/- 0.07) | 0.97 (+/- 0.07) | 0.97 (+/- 0.06) |
| Meta - Quadratic Discriminant Analysis | 0.49 (+/- 0.02) | 0.00 (+/- 0.00) | 0.00 (+/- 0.00) | 0.00 (+/- 0.00) |
| Meta - Nearest Centroid | 0.97 (+/- 0.06) | 0.98 (+/- 0.07) | 0.97 (+/- 0.07) | 0.97 (+/- 0.06) |
| Meta - Bernoulli NB | 0.97 (+/- 0.06) | 0.97 (+/- 0.08) | 0.98 (+/- 0.05) | 0.97 (+/- 0.06) |
| Meta - Gaussian NB | 0.97 (+/- 0.06) | 0.97 (+/- 0.08) | 0.98 (+/- 0.05) | 0.97 (+/- 0.06) |
| Meta - Dummy Classifier | 0.51 (+/- 0.02) | 0.51 (+/- 0.02) | 1.00 (+/- 0.00) | 0.67 (+/- 0.02) |

Table 8. Comparison results of the proposed method with previous works (TUH EEG corpus dataset)

| Feature | Model | Dataset | Result | Reference |
| --- | --- | --- | --- | --- |
| Spectrogram | Convolutional neural network | TUH | Acc. 88.30% | [45] |
| Automated identification without feature extraction | One dimensional deep convolutional neural network | TUH | Acc. 79.34% | [39] |
| 32 Features extracted from EEG signals | Base-2-Meta | TUH | Acc. 98% | This work |
